# Short-Term Effect of Conventional Versus Accelerated Corneal Cross-Linking Protocol on Corneal Geography and Stability

**DOI:** 10.3390/medicina59061043

**Published:** 2023-05-28

**Authors:** Sania Vidas Pauk, Sonja Jandroković, Dina Lešin Gaćina, Martina Tomić, Tomislav Bulum, Ana Pupić Bakrač, Tomislav Kuzman, Josip Knežević, Miro Kalauz

**Affiliations:** 1 Department of Ophthalmology, Zagreb University Hospital Center, 10000 Zagreb, Croatia; 2 School of Medicine, University of Zagreb, Šalata 2, 10000 Zagreb, Croatia; 3 Vuk Vrhovac University Clinic for Diabetes, Endocrinology and Metabolic Diseases, Merkur University Hospital, Dugi dol 4a, 10000 Zagreb, Croatia

**Keywords:** keratoconus, corneal crosslinking, conventional CXL, accelerated CXL, ABCD grading system, tomography

## Abstract

*Purpose*: To determine the 6-month effect of conventional (CXL30) and accelerated cross-linking with a UVA intensity of 9 mW/cm^2^ (CXL10) on corneal stability and to investigate whether there was a difference in ABCD grading system parameters regarding the two different procedures. *Methods*: Twenty-eight eyes of 28 patients with a documented keratoconus (KN) progression were included. Patients were selected to undergo either epi off CXL30 or CXL10. At the baseline and the follow-up visits after one (V1), three (V2), and six months (V3), the patients underwent complete ophthalmic examination and corneal tomography. *Results*: In the CXL30 group, all the parameters from the ABCD grading system significantly changed from baseline to V3; parameter A decreased (*p* = 0.048), B and C increased (*p* = 0.010, *p* < 0.001), and D decreased (*p* < 0.001). In the CXL10 group, there were no changes in parameters A (*p* = 0.247) and B (*p* = 0.933), though parameter C increased (*p* = 0.001) and D decreased (*p* < 0.001). After an initial decline after one month, visual acuity (VA) recovered on V2 and V3 (*p* < 0.001), and median maximal keratometry (Kmax) decreased in both groups (*p* = 0.001, *p* = 0.035). In the CXL30 group, there were significant changes in other parameters; average pachymetric progression index (*p* < 0.001), Ambrósio relational thickness maximum (ARTmax) (*p* = 0.008), front and back mean keratometry (*p* < 0.001), pachymetry apex (PA) (*p* < 0.001), and front elevation (*p* = 0.042). However, in the CXL10 group, there were significant changes only in ARTmax (*p* = 0.019) and PA (*p* < 0.001). *Conclusion*: Both epi-off CXL protocols showed similar short-term efficacy in improving VA and Kmax, halting the progression of KN, and both similarly changed tomographic parameters. However, the conventional protocol modified the cornea more significantly.

## 1. Introduction

Corneal cross-linking (CXL) is well-established, widely used, and the only approved method to slow and stop the progression of keratoconus (KN) and has been showing promising results for over a decade or more [1,2,3,4,5,6,7,8]. CXL increases the stiffness and rigidity of the cornea by photochemical cross-linking of the corneal collagen fibers using a combination of riboflavin and ultraviolet A (UVA) radiation [9,10,11,12]. The conventional CXL protocol was first introduced in a clinical setting by Wollensak and coworkers [4]. It comprises 30 min of UVA illumination of 3 mW/cm^2^ irradiance with 370 nm light with a cumulative dose of 5.4 J/cm^2^ onto the cornea. Recently, many authors introduced new protocols for CXL, relying on the Bunsen–Roscoe law of reciprocity [13,14,15,16,17,18,19,20,21,22,23,24,25,26,27]. The largest number of different CXL procedures, regardless of whether performed in epi-off or in epi-on, are based on empirical concepts such as the 3 mW/cm2 of intensity performed in the Dresden protocol and all the accelerated ones using the application of Bunsen Roscoe’s law. This law dates back to the 1800s and is valid for transparent solid bodies, but it has yet to be demonstrated whether it also works on transparent biological tissues such as the cornea. The only protocol that is not based on empirical concepts is the custom fast CXL which, instead, is based on published experimental data which have allowed us to know (1) the average concentration of riboflavin present in the corneal stroma after imbibition and (2) the rate of consumption of riboflavin under the effect of UVA during the irradiation phase of the CXL. All this has allowed the creation of a mathematical algorithm whereby entering the corneal thinnest point, the Kmax, and the coordinates of the corneal apex, it calculates exactly the energy and intensity of the UVA beam to be used in a CXL procedure for that specific affected patient from keratoconus. Furthermore, the protection of the endothelium is guaranteed because the known threshold limit of 0.35 mW/cm^2^ at the endothelial level is never exceeded. This is why this procedure is called customized.

The epi-off method that uses a UVA intensity of 9 mW/cm^2^ with 10 min of exposure time shows comparable clinical outcomes in stopping the progression of KN, remodeling the cornea, and restoring the visual function compared to the Dresden protocol in many studies [13,14,15,16,17,18,19]. Only two studies presented better clinical improvement with more significant corneal flattening in patients undergoing conventional CXL [28,29]. The efficacy of those two protocols was mainly evaluated and compared by measuring the changes in the anterior surface keratometry readings, visual function, and manifest refraction 6 to 24 months after the procedures and the demarcation line depth one month postoperatively. However, the results on the depth of the demarcation line 1 month after performing the two protocols were inconsistent in various studies. Kymionis et al. and Tomita et al. found a similar demarcation line depth between 9 mW/cm^2^ and the conventional protocol when using dextran–riboflavin [30,31]. Hagem and coauthors showed an even deeper demarcation line in the 9 mW/cm^2^ accelerated protocol compared to the traditional protocol when methylcellulose–riboflavin was applied in both groups [14]. On the contrary, Shajari and coauthors, in their meta-analysis, reported a significantly shallower demarcation line in the 9 mW/cm^2^ protocol [17]. These findings raise the concern of whether the efficacy of different CXL procedures can be objectively obtained only by evaluating the anterior corneal surface parameters, mostly apically based, visual acuity, and refractive error. It is also concerning whether the results of different studies are comparable, counting the heterogeneity of methodologies, exposure times, different time intervals, total instillation times, solutions used, and the paucity of randomized clinical trials.

The endothelial cell density and complication rate, which was low after the two procedures, were comparable 6–24 months postoperatively, pointing to both methods being equally safe [13,14,15,17]. 

A newly developed classification system, the ABCD keratoconus grading system introduced by Belin and coworkers in 2016, incorporated into the Scheimpflug tomography device (Pentacam HR; OCULUS Optikgeräte GmbH, Wetzlar, Germany), brought lots of improvements in diagnosing of KN [32]. The system graduates KN according to the three machine-generated parameters, the anterior and posterior radii of curvature taken from a 3-mm zone centered on the thinnest point and minimum corneal thickness, and the fourth aspect is estimated on the base of the user-entered patient visual function. Previous classification systems have severe limitations in ignoring the posterior corneal surface. Their measurements are apically based, ignoring the actual thinnest point on the cornea, and, as such, are not centered on the cone [8,33,34]. It is well-known that early KN presents with changes in the posterior cornea before the anterior surface changes [35]. In addition, apical-based anterior surface measurements may not reflect the actual changes caused by various CXL protocols since it is speculated that the cone location affects the amount of corneal flattening after the procedure [36,37]. The ABCD system analyzes the anterior and posterior corneal surface in the cone location and combines the visual function in the final gradation [32]. That exceeds the limitations of previous systems and leads to a more accurate evaluation of KN, disease progression, and CXL results and potentially might be more accurate for investigating how different CXL protocols affect corneal parameters and stability.

Therefore, the present study aimed to determine the early, short-term, and 6-month effects of conventional CXL (CXL30) and ACXL–UVA 9 mW/cm^2^ (CXL10) epi-off procedures on corneal stability and visual function and to investigate whether there was a difference in ABCD grading system parameters regarding the two different procedures.

## 2. Methods

### 2.1. Ethics

This prospective, longitudinal study was performed in the tertiary-eye institution, Department of Ophthalmology, University Clinical Hospital Center Zagreb, following the Declaration of Helsinki and was approved by the Hospital’s Ethics Committee. It took place from May 2021 to May 2022. The patients received written and oral information about the intervention and study and signed written informed consent.

### 2.2. Subjects and Study Details

The study included twenty-eight eyes of 28 patients. Eligible subjects had to have a documented diagnosis of keratoconus progression, corneal thickness 370 um or more measured with Scheimpflug imaging (Pentacam HR; Oculus, Wetzlar Inc, Germany), no central corneal scars, chemical burns, severe corneal infection, or ocular surface disease, or pregnancy and lactation during treatment. A corneal expert and the first author assessed the diagnosis and monitoring for disease progression. Progression of the KN was defined following the instructions from the Global Consensus on Keratoconus and Ectatic Diseases [38]. “Ectasia progression” is defined by a consistent change in at least 2 of the following parameters where the magnitude of the change is above the normal noise of the testing system: 1. Steepening of the anterior corneal surface 2. Steepening of the posterior corneal surface 3. Thinning and/or an increase in the rate of corneal thickness change from the periphery to the thinnest point.” The changes need to be consistent over time and above the normal variability (i.e., noise) of the measurement system (this will vary by the system).” After individual determination of each patient to have disease progression, patients were selected to undergo either a conventional (for 30 min (CXL30 group)) or accelerated (for 10 min (CXL10 group)) epi-off procedure.

Before the procedure, the eye was anesthetized with 1% tetracaine, and 1% pilocarpine was used to constrict the pupil. Under sterile conditions, the surgeon mechanically removed the corneal epithelium using a blunt hockey knife and presoaked the cornea with riboflavin 0.1% with hydroxypropyl methylcellulose 1.1% (MedioCROSS M, MedioCROSS, Medio-Haus-Medizinprodukte GmbH Kiel, Germany) for 10 min in 2-min intervals. The CXL30 and CXL10 groups were irradiated with UVA light at the irradiance of 3 mW/cm^2^ for 30 min and irradiance of 9 mW/cm^2^ for 10 min, respectively. The methylcellulose–riboflavin solution was applied every 5 min during UVA irradiation. After the procedure, the cornea was rinsed with a balanced salt solution, double-antibiotic and dexamethasone were applied topically, and a bandage soft contact lens (CL) was inserted and used for six days. Postoperatively, preservative-free topical antibiotics (moxifloxacin) and tobramycin-dexamethasone therapy were recommended for up to six days. Afterward, CL was removed and patients were instructed to continue with tobramycin–dexamethasone with gradual tapering. When finished, the patients proceeded with preservative-free dexamethasone once to twice daily for one month.

On the day of the procedure, the baseline visit (V0), and on all the follow-up visits, after one month (V1), three months (V2), and six months (V3) postoperatively, the patients underwent complete ophthalmic examination, including uncorrected visual acuity testing (UDVA), best-corrected visual acuity testing (BCVA), comprehensive slit-lamp examination, and corneal tomography (Pentacam HR; Oculus, Wetzlar Inc, Germany). All corneal imaging was performed by the first author and rechecked by the third. Patients were instructed not to wear CL for 5–7 days before the visits.

### 2.3. Statistical Analyses

Statistical analysis was performed using the Statistica software package version 14.0.1. (TIBCO Inc., Palo Alto, CA, USA)Continuous data were presented as mean ± SD or median (min-max), and categorical data as numbers (percentages). After testing the normality of data distribution by the Shapiro–Wilk test, the appropriate parametric and nonparametric tests were used. For continuous data, differences between two independent groups were tested by *t*-test or Mann–Whitney test, and between multiple dependent variables by repeated measures ANOVA test or Friedman ANOVA test. The Scheffe test and Wilcoxon test were used for post hoc analyses. For categorical data testing, the Chi-square test was used. A *p*-value of less than 0.05 was considered statistically significant in all analyses.

## 3. Results

Twenty-eight eyes of 28 patients (24 males/4 females, mean age 24.86 ± 5.70 years) treated by CXL for keratoconus progression were included in this prospective study and followed for six months. According to the CXL protocol, patients were divided into two groups: the CXL30 group (*n* = 15)—patients who underwent conventional CXL for 30 min, and the CXL10 group (*n* = 13)—patients who underwent accelerated CXL for 10 min.

**Figure 1:**
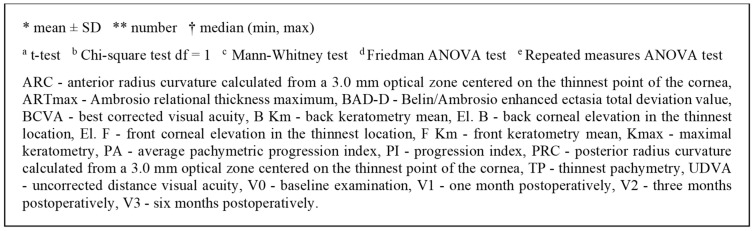
A legend indicating all the symbols and abbreviations, here listed in alphabetical order, used in the tables, with the statistical tests performed.

Figure 1 presents a legend indicating all the symbols and abbreviations used in the tables, with the statistical tests performed. Abbreviations are here listed in alphabetical order.

The baseline characteristics of patients (eyes) included in the study and divided into two CXL groups are presented in Table 1. Patients in the CXL30 group were significantly younger than those in the CXL10 group (*p* = 0.003), but no significant differences in gender, side of the eye (right or left), Kmax, severity of KN, uncorrected distance and best corrected visual acuity, and refractive errors in sphere or cylinder were found between the groups (*p* > 0.05).

In both groups, the median of UDVA and BCVA significantly increased (*p* < 0.001), while Kmax significantly decreased (CXL30 *p* = 0.001; CXL10 *p* = 0.035) from baseline to study visits (per-protocol set) (Table 2). For the UDVA, the post hoc analysis showed a significant increase in the CXL30 group from the V2 to the V3 (Z = 2.666, *p* = 0.008) and in the CXL10 group from the V1 to the V2 (Z = 2.366, *p* = 0.018), while for the BCVA, a significant increase in both groups was observed from the V1 to the V2 (CXL30 Z = 2.667, *p* = 0.008; CXL10 Z = 2.521, *p* = 0.012), and in the CXL10 group also from the V2 to the V3 (Z = 2.132, *p* = 0.033). Contrary to the previous results, after an initial significant increase in both groups from the V0 to the V1 (CXL30 Z = 2.489, *p* = 0.028; CXL10 Z = 2.097, *p* = 0.036), Kmax significantly decreased in the CXL30 group from the V1 to the V2 (Z = 1.977, *p* = 0.048) and in CXL10 group from the V2 to the V3 (Z = 2.731, *p* = 0.006) 

**Figure 2:**
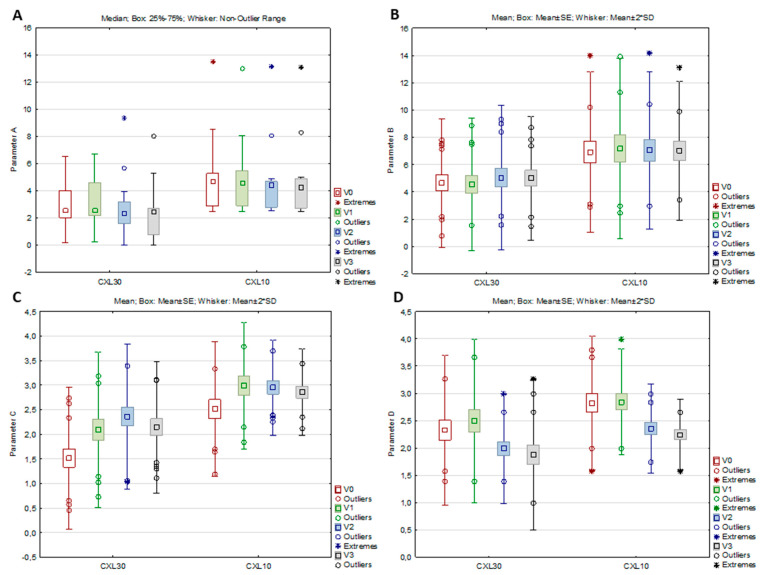
Parameters A (**A**), B (**B**), C (**C**), and D (**D**) of the CXL30 and CXL10 group at the baseline visit (V0), one (V1), three (V2), and six months (V3) after the corneal cross-linking procedure.

In the CXL 30 group, all parameters from the ABCD grading system, as well as the anterior radius of curvature (ARC), the posterior radius of curvature (PRC), and thinnest pachymetry (TP), significantly changed from baseline to study visits (per-protocol set), while in the CXL 10 group, significant changes were observed only in parameter C, parameter D, and TP (Table 3, Figure 2A–D). According to the post hoc analysis, parameter A decreased significantly in the CXL30 group from V2 to V3 (Z = 2.551, *p* = 0.011), parameter B increased significantly in the same group from V1 to V2 (*p* = 0.027), while parameter C in both groups increased significantly from V0 to V1 (CXL30 *p* < 0.001; CXL10 *p* = 0.006) and parameter D decreased significantly from V1 to V2 (CXL30 *p* < 0.001; CXL10 *p* = 0.016)). For the ARC and PRC in the CXL30 group, the post hoc analysis showed a significant increase of ARC from the V2 to the V3 (*p* = 0.041) and a significant decrease of PRC from the V2 to the V3 (*p* = 0.020), while for the TP, a significant decrease in both groups was found from the V0 to the V1 (CXL30 *p* < 0.001; CXL10 *p* = 0.042) 

In the CXL30 group, the other analyzed variables, Belin/Ambrósio enhanced ectasia total deviation value (BAD-D), average pachymetric progression index (PI), Ambrósio relational thickness maximum (ARTmax), front keratometry mean (F Km), back keratometry mean (B Km), pachymetry apex (PA), and back elevation in the thinnest location (El. B), also changed significantly from baseline to study visits (per-protocol set), whereas, in the CXL 10 group, the significant changes were found only in the ARTmax and PA (Table 4). According to the post hoc analysis, all significant changes in the CXL30 group occurred from V1 to V2, BAD-D (Z = 2.354, *p* = 0.019), and El. B significantly increased (*p* = 0.046), while PI (*p* < 0.001), F Km (*p* = 0.001), and B Km (*p* = 0.002) significantly decreased (not table shown data). ARTmax and PA significantly decreased in both groups from V0 to V1 (ARTmax CXL30 *p* = 0.016, CXL10 *p* = 0.030; PA CXL30 *p* < 0.001, CXL10 *p* = 0.005) (not table shown data). Front elevation in the thinnest location (EL. F) did not significantly change throughout the study visits in both groups (*p* > 0.05).

## 4. Discussion

This study investigated the difference between two epi-off CXL protocols, conventional and ACXL, with a UVA intensity of 9 mW/cm^2^ on early, six-month outcomes, corneal stability, and visual acuity. Although many other studies already showed comparable one- or two-year results of those two protocols, the main advantage of this study is that besides analyzing parameters from the anterior corneal surface in the center of the cornea solely, together with visual acuity and refractive errors, it was the first, to our knowledge, that analyzed parameters from the anterior and posterior corneal surface in the center of the cone together with corneal thickness changes and their relation regarding the two protocols [13,14,15,16,17,18,19]. It brought a deeper analysis of corneal changes in the early postoperative phase, which could help better in understanding how different protocols affect corneal parameters and stability.

The present study results showed comparable and significant improvements in visual acuity and maximal keratometry readings in both groups of patients regarding both CXL30 and CXL10 protocols. In both groups, after initial deterioration at one month, both parameters significantly improved after three and six months, which is comparable with other study results [13,14,15,16,17,18,19]. It may be assumed that both protocols presented similar efficacy in clinical outcomes and halting the progression of KN in the early postoperative, six-month phase.

On the contrary, a significant decline in parameter A was only observed in the CXL30 group, while in the CXL10 group, there were no significant changes in that parameter. However, after the initial tendency of increasing one month postoperatively, there was little tendency to decrease parameter A in the latter group after three and six months. On the other hand, front corneal elevation in the thinnest location did not show significant changes in both groups, showing a similar decline tendency after three and six months. After initial steepening, the CXL30 protocol resulted in more significant flattening of the anterior corneal surface in the 3 mm cone center zone three and six months postoperatively, while CXL10 did not change the anterior corneal surface significantly. It only showed a small tendency to flatten that zone. Parameter B increased significantly in the CXL30 group, while in the CXL10 group, there were no significant changes in that parameter. Similar to parameter A, there was also a small tendency for an initial increase of parameter B in the CXL10 group, which showed a tendency to decrease after three and six months. Similar changes were observed in back corneal elevation in the thinnest location, which significantly increased only in the CXL30 group, while in the CXL10 group, there were no changes in the back elevation. The CXL30 protocol resulted in a significant steepening of the back corneal surface in the center of the cone, which was most pronounced at one month, with the tendency of flattening after three and six months. On the contrary, the CXL10 protocol did not significantly change the corneal surface in the cone center, although there was little tendency for initial steepening and later flattening. Parameter C increased significantly in both groups, with the tendency to decrease after six months postoperatively. Both groups observed similar changes in ART max, while PI only significantly changed in the CXL30 group. These results showed that both protocols significantly thinned the cornea in the early postoperative phase. However, at the end of the six-month follow-up period, parameters on corneal pachymetry showed a tendency for improvement, which would have been displayed in the further postoperative period if it had been longer. After an initial increase at one month, parameter D significantly decreased in both groups of patients, showing that after the initial decline of vision quality after one month, both groups of patients experienced a significant functional visual recovery tree six months after treatment.

In the literature, only a few studies evaluated the applicability of the ABCD staging system in monitoring the efficacy of the standard procedure. In contrast, only one recently published retrospective study investigated its utility in evaluating accelerated protocols and compared the effectiveness of different protocols regarding the ABCD grading system [8,39,40,41,42,43]. Danesh and coauthors showed no significant changes in parameters A and D, while parameters B and C significantly increased one year after conventional CXL [8]. Krolo et al. investigated the efficacy of the standard protocol according to the cone location, central or peripheral. They showed no significant changes in parameter A and a significant increase in parameters B and C in the 12-month post-treatment period [41]. Parameter D initially increased and returned to the preoperative value one year after the standard CXL procedure. The presented studies showed similar results to the herein presented study, except for the difference in changes of parameters A in the CXL30 group and D in both groups, which showed significant improvement in the present study, contrary to the previously mentioned. A possible explanation might be the difference in the severity of KN in the studies. In the present study, median maximal keratometry readings were 57.2 and 60 D in CXL30 and CX10 groups, while in the former two studies, they were 52.3 and 52.9, meaning that subjects in two other studies had milder diseases than in our study. In their research, Greenstein and Hersh showed better visual function recovery in more advanced KN after CXL [44]. Saglik et al. found a regression in parameter A in the one-year post-standard procedure follow-up, as in the CXL30 group in the present study. Other parameters showed similar trends to the current study [39]. However, they found a significant positive correlation between the change in Kmax values and parameter A. Similar to Saglik and coauthors, Bardan et al. found a reduction of parameter A six and 12 months postoperatively [40]. Contrary, there were no significant changes in parameter B. Parameter C significantly progressed while D remained stable one year after the procedure. Recently, in 2023, a retrospective study was published, the first to our knowledge to evaluate the efficacy of five different accelerated protocols regarding the ABCD staging system one-year post-treatment [43]. The results showed that parameters A and B decreased in the Iontophoresis CXL group. Parameter A significantly reduced in the High-Fluence Accelerated CXL group. However, parameters B and C increased significantly in the High-Fluence Accelerated CXL and CXL-plus-PTK groups.

Currently, the ABCD grading system is recognized as more appropriate for diagnosing, staging, and monitoring KN as well as evaluating the effectiveness of CXL on corneal changes and halting the progression than keratometry readings, which cannot provide information on morphological changes of the entire cornea [8]. However, studies investigating the efficacy of different protocols according to the previously mentioned more accurate grading system are lacking.

In the current study, the conventional and epi-off ACXL with a UVA intensity of 9 mW/cm^2^ protocols showed similar efficacy in early postoperative clinical and structural outcomes. However, the CXL30 protocol resulted in more significant structural and geographical changes. More studies will be necessary with longer follow-ups to further clarify these findings in terms of the long-term halting of KN progression and clinical outcomes. Additionally, this study confirmed the importance of utilizing the ABCD keratoconus grading system in evaluating the efficacy of different CXL procedures since it might provide more profound information on how different procedures change corneal stability and geography and indicate and predict long-term effects. At a time when numerous protocols are available, a better understanding of how each of them changes the cornea could help clinicians choose the right one for a particular patient.

## 5. Conclusions

This study investigated the efficacy of conventional versus epi-off ACXL with a UVA intensity of 9 mW/cm2 protocols on clinical and structural outcomes in the cornea and the capability of halting the progression of keratoconus. However, while traditional parameters used for evaluation of effectiveness, e.g., visual function, refractive errors, and parameters from the anterior corneal surface (Kmax), showed very good and comparable results in both protocols, the ABCD grading system, as well as other parameters from the anterior and posterior corneal surface in the cone center and corneal thickness, showed more significant changes in the patients who underwent the standard procedure. Therefore, it is essential to incorporate the ABCD staging system into the assessment and comparison of the efficacy of various CXL protocols to understand and clarify better how different procedures affect the corneal structure and geography and to investigate whether those parameters might indicate and predict long-term effects that, in future, might help clinicians choose the suitable protocol for their patients.

## Figures and Tables

**Table 1:** Baseline characteristics of 28 patients (eyes) included in the study and divided into two groups according to the CXL protocol.

	CXL30 (n = 15)	CXL10 (n = 13)	t ^a^ χ^2 b^ Z ^c^	*p*
Age (years) *	22.58 ± 4.48	28.08 ± 5.27	−2.797 ^a^	0.010
Gender (m/f) **	9/3	12/1	1.391 ^b^	0.238
Eye (right/left) **	8/7	7/6	0.001 ^b^	0.978
Kmax ≥ 55, BCVA ≤ 0.5/Kmax < 55, BCVA > 0.5 **	12/3	13/0	2.912 ^b^	0.088
Kmax †	57.2 (47–77)	60 (53–90)	−1.750 ^c^	0.080
UDVA (decimal) †	0.2 (0.05–0.8)	0.1 (0.01–0.5)	1.727 ^c^	0.084
Sphere (dptr) †	−1.00 (0.00, −7.00)	−1.50 (0.50, −14.00)	0.668 ^c^	0.504
Cylinder (dptr) †	−1.25 (0.00, −5.00)	−2.50 (0.00, −6.00)	1.543 ^c^	0.123
BCVA (decimal) †	0.4 (0.15–0.8)	0.2 (0.08–0.7)	1.589 ^c^	0.112

* mean ± SD; ** number; † median (min, max); a: *t*-test; b: Chi-square test df = 1; c: Mann-Whitney test.

**Table 2:** Visual acuity of the CXL30 and CXL10 group at the baseline visit, one, three, and six months after the corneal cross-linking procedure.

**UDVA** **†**						
	V0	V1	V2	V3	χ^2 d^	*p*
CXL30	0.2 (0.05–0.8)	0.3 (0.05–0.7)	0.4 (0.05–0.7)	0.5 (0.05–1.0)	17.388	**<0.001**
CXL10	0.1 (0.01–0.5)	0.1 (0.03–0.3)	0.2 (0.08–0.5)	0.2 (0.10–0.6)	16.885	**<0.001**
**BCVA** **†**						
	V0	V1	V2	V3	χ^2 d^	*p*
CXL30	0.4 (0.15–0.8)	0.3 (0.10–0.8)	0.6 (0.20–0.8)	0.6 (0.15–1.0)	24.529	**<0.001**
CXL10	0.2 (0.08–0.7)	0.3 (0.05–0.5)	0.4 (0.20–0.6)	0.4 (0.30–0.7)	18.031	**<0.001**
**Kmax** **†**						
	V0	V1	V2	V3	χ^2 d^	*p*
CXL30	57.2 (47–77)	57.9 (48–80)	57.6 (46–76)	55.4 (46–76)	15.919	**0.001**
CXL10	60 (53–90)	63 (55–87)	60 (54–87)	60 (54–89)	8.615	**0.035**

† median (min, max); d—Friedman ANOVA test.

**Table 3:** The ABCD grading system parameters, ARC, PRC, and TP of the CXL30 and CXL10 group at the baseline visit, one, three, and six months after the corneal cross-linking procedure.

**Parameter A** **†**						
	V0	V1	V2	V3	χ^2 d^	*p*
CXL30	2.6 (0.2–6.5)	2.6 (0.3–6.7)	2.4 (0–9.4)	2.5 (0.2–8.0)	7.905	0.048
CXL10	4.7 (2.5–13.5)	4.6 (2.5–13.0)	4.4 (2.5–13.2)	4.3 (2.5–13.1)	4.139	0.247
Parameter B *						
	V0	V1	V2	V3	F ^e^	*p*
CXL30	4.66 ± 2.34	4.54 ± 2.43	5.03 ± 2.65	4.99 ± 2.26	4.292	0.010
CXL10	6.92 ± 2.94	7.18 ± 3.31	7.05 ± 2.89	7.01 ± 2.55	0.144	0.933
Parameter C *						
	V0	V1	V2	V3	F ^e^	*p*
CXL30	1.52 ± 0.72	2.09 ± 0.79	2.36 ± 0.74	2.14 ± 0.67	25.776	<0.001
CXL10	2.51 ± 0.69	2.98 ± 0.64	2.95 ± 0.48	2.86 ± 0.44	6.847	0.001
Parameter D *						
	V0	V1	V2	V3	F ^e^	*p*
CXL30	2.33 ± 0.69	2.49 ± 0.75	1.99 ± 0.50	1.87 ± 0.69	17.629	<0.001
CXL10	2.82 ± 0.61	2.84 ± 0.48	2.35 ± 0.41	2.24 ± 0.32	12.839	<0.001
ARC *						
	V0	V1	V2	V3	F ^e^	*p*
CXL30	6.62 ± 0.54	6.59 ± 0.54	6.73 ± 0.73	6.85 ± 0.54	3.995	0.014
CXL10	5.97 ± 0.66	5.96 ± 0.66	6.05 ± 0.62	6.06 ± 0.64	1.859	0.158
PRC *						
	V0	V1	V2	V3	F ^e^	*p*
CXL30	4.89 ± 0.58	4.92 ± 0.58	4.81 ± 0.62	4.64 ± 0.84	4.056	0.013
CXL10	4.37 ± 0.59	4.33 ± 0.69	4.34 ± 0.58	4.34 ± 0.51	0.058	0.982
TP *						
	V0	V1	V2	V3	F ^e^	*p*
CXL30	470.60 ± 34.82	442.00 ± 37.93	428.33 ± 36.95	439.47 ± 32.13	21.936	<0.001
CXL10	419.00 ± 31.20	396.82 ± 55.58	393.38 ± 33.57	402.15 ± 27.37	3.590	0.025

* mean ± SD; † median (min, max); d—Friedman ANOVA test; e—Repeated measures ANOVA test.

**Table 4:** Corneal parameters and indices of progression of the CXL30 and CXL10 group at the baseline visit and one, three, and six months after the corneal cross-linking procedure.

**BAD-D** **†**						
	V0	V1	V2	V3	χ^2 d^	*p*
CXL30	9 (1.5–15.5)	8.6 (1.6–18.6)	9.3 (2.2–20.9)	9.6 (4.1–18.3)	16.200	0.001
CXL10	13.0 (8–33.7)	13.2 (8–34.3)	13.5 (9–33.0)	13.1 (8–31.6)	2.119	0.548
PI *						
	V0	V1	V2	V3	F ^e^	*p*
CXL30	2.15 ± 0.62	2.46 ± 0.78	2.78 ± 0.83	2.81 ± 0.66	9.161	<0.001
CXL10	2.92 ± 0.85	3.57 ± 1.58	3.43 ± 1.17	3.25 ± 0.79	2.483	0.079
ARTmax *						
	V0	V1	V2	V3	F ^e^	*p*
CXL30	156.0 ± 50.8	125.8 ± 50.9	119.6 ± 40.7	124.0 ± 38.9	4.531	0.008
CXL10	110.4 ± 46.0	86.7 ± 50.0	90.8 ± 30.6	92.7 ± 28.3	3.829	0.019
F Km *						
	V0	V1	V2	V3	F ^e^	*p*
CXL30	48.12 ± 3.38	48.90 ± −3.96	47.84 ± 4.25	46.97 ± 4.01	6.703	<0.001
CXL10	48.56 ± 15.48	53.43 ± 6.49	52.16 ± 6.22	51.95 ± 6.54	0.958	0.425
B Km *						
	V0	V1	V2	V3	F ^e^	*p*
CXL30	−7.21 ± 0.75	−7.09 ± 0.75	−7,36 ± 0.79	−7.38 ± 0.76	9.249	<0.001
CXL10	−8.00 ± 1.26	−8.13 ± 1.46	−8.08 ± 1.35	−8.01 ± 1.17	1.058	0.382
PA *						
	V0	V1	V2	V3	F ^e^	*p*
CXL30	482.80 ± 35.29	459.07 ± 32.14	446.00 ± 30.58	451.73 ± 27.49	22.345	<0.001
CXL10	432.92 ± 32.44	402.45 ± 44.64	405.77 ± 35.35	416.08 ± 30.24	7.386	<0.001
El. F *						
	V0	V1	V2	V3	F ^e^	*p*
CXL30	24.87 ± 11.47	26.43 ± 9.38	23.87 ± 15.09	21.33 ± 15.45	1.243	0.307
CXL10	36.38 ± 17.75	36.00 ± 18.55	34.23 ± 16.80	34.54 ± 17.38	0.395	0.758
El. B *						
	V0	V1	V2	V3	F ^e^	*p*
CXL30	55.67 ± 24.90	55.86 ± 22.62	58.13 ± 25.35	60.27 ± 23.86	2.991	0.042
CXL10	77.15 ± 33.37	77.73 ± 40.06	77.08 ± 35.63	78.54 ± 31.14	0.143	0.933

* mean ± SD; † median (min, max); d—Friedman ANOVA test; e—Repeated measures ANOVA test.

## Data Availability

The authors confirm that the data supporting the findings of this study are available within the article, and from the corresponding author upon request.
